# Movement variability in stroke patients and controls performing two upper limb functional tasks: a new assessment methodology

**DOI:** 10.1186/1743-0003-6-2

**Published:** 2009-01-23

**Authors:** Sibylle B Thies, Phil A Tresadern, Laurence P Kenney, Joel Smith, David Howard, John Y Goulermas, Christine Smith, Julie Rigby

**Affiliations:** 1 Centre for Rehabilitation and Human Performance Research, University of Salford, Salford, Greater Manchester, UK; 2 Department of Electrical Engineering and Electronics, University of Liverpool, Liverpool, UK

## Abstract

**Background:**

In the evaluation of upper limb impairment post stroke there remains a gap between detailed kinematic analyses with expensive motion capturing systems and common clinical assessment tests. In particular, although many clinical tests evaluate the performance of functional tasks, metrics to characterise upper limb kinematics are generally not applicable to such tasks and very limited in scope. This paper reports on a novel, user-friendly methodology that allows for the assessment of both signal magnitude and timing variability in upper limb movement trajectories during functional task performance. In order to demonstrate the technique, we report on a study in which the variability in timing and signal magnitude of data collected during the performance of two functional tasks is compared between a group of subjects with stroke and a group of individually matched control subjects.

**Methods:**

We employ dynamic time warping for curve registration to quantify two aspects of movement variability: 1) variability of the timing of the accelerometer signals' characteristics and 2) variability of the signals' magnitude. Six stroke patients and six matched controls performed several trials of a unilateral ('drinking') and a bilateral ('moving a plate') functional task on two different days, approximately 1 month apart. Group differences for the two variability metrics were investigated on both days.

**Results:**

For 'drinking from a glass' significant group differences were obtained on both days for the timing variability of the acceleration signals' characteristics (p = 0.002 and p = 0.008 for test and retest, respectively); all stroke patients showed increased signal timing variability as compared to their corresponding control subject. 'Moving a plate' provided less distinct group differences.

**Conclusion:**

This initial application establishes that movement variability metrics, as determined by our methodology, appear different in stroke patients as compared to matched controls during unilateral task performance ('drinking'). Use of a user-friendly, inexpensive accelerometer makes this methodology feasible for routine clinical evaluations. We are encouraged to perform larger studies to further investigate the metrics' usefulness when quantifying levels of impairment.

## Background

Stroke affects approximately 2 in 1000 people in the UK per year [1] and impaired upper limb function is reported to be a major problem [2]. At 3 months post stroke only 20% of patients have normal upper limb function [3] and less than 15% with initial paralysis may regain complete motor recovery [4]. Although there exist a number of promising approaches to the promotion of upper limb recovery after stroke, quantifying the effectiveness of such interventions remains somewhat limited by the available outcome measures.

Previous research found that following stroke upper limb movement trajectories during point-point reaching are more spatially segmented and motions are performed at slower speeds and with greater trunk involvement as compared to healthy controls [5]. Furthermore, upper limb movement smoothness during reaching, as characterized by jerk, has shown good correlation with stroke recovery [6]. Although these studies provided valuable insights into how stroke affects upper limb kinematics, only the forward reach and retraction of the arm during pointing tasks were investigated with expensive equipment such as 3D camera motion analysis systems that cannot easily be moved within the clinic or to a patient's home.

At present there remains a gap between such objective kinematic measures of upper limb impairment which characterise non-functional tasks (e.g. pointing tasks) in great detail [5-8] and clinical measures that evaluate functional task performance. Clinical tests often measure the time to complete a certain task (e.g. box-and-blocks test) [9], or collect categorical measurements of performance (e.g. ARAT) [9]. Others, for example the Motricity Index [9], evaluate impairment quantitatively, however, previous work has addressed limitations of such tests, for example, poor standardization and/or reliability [10-13].

We therefore developed a new methodology for the characterization of functional upper limb movements which could bridge the gap between clinical assessment tests and complex, objective kinematic description of non-functional pointing tasks. More specifically, we employed user-friendly, inexpensive accelerometers for which we see many advantages in routine clinical evaluations. A small number of studies [14-16] have recently made use of inertial sensor technology to describe upper limb kinematics in functional tasks but have yet to develop appropriate metrics to characterise the motions.

**Figure 1:**
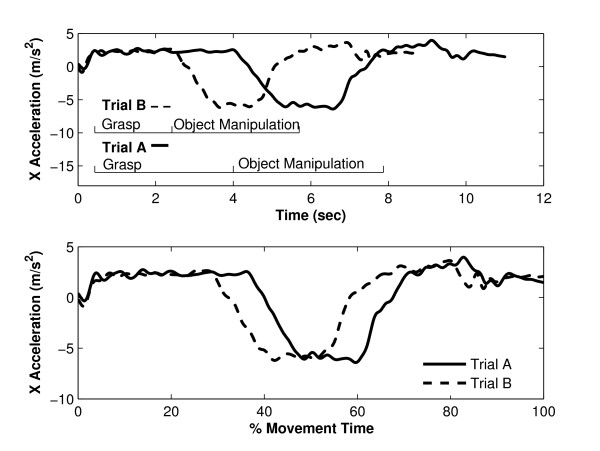
**Application of time normalization to upper limb kinematics**. Presentation of kinematic data from two repeats of a functional upper limb task (top) and illustration of the effect that uniform normalization of the time axis has on the data (bottom).

Standard approaches to movement variability quantification in upper limb movements are typically based on the spread in the value of characteristic features, such as peak velocity, or end point error in pointing tasks [5]. For gait data, Chau [17] makes a strong case for considering variability across the entire curves, rather than variability in the magnitude of particular, discrete features. Chau and others also identify that random noise and phase variation between trials suggests the use of more sophisticated approaches than time normalisation and simple descriptive statistics when comparing motion curves[17,18]. Clearly, for upper limb functional tasks, in which the duration of each part of the movement (e.g. reach, manipulate, release) is likely to vary both within and between individuals, time normalisation introduces the risk of aligning trials inappropriately. For example, consider two trials of a functional upper limb task in which the subject took significantly longer to complete the grasp of the object in Trial A, as compared to Trial B (Figure 1, top). By linearly compressing signals, it is highly likely that data points from one part of the task gathered during Trial A could be compared with data from a completely different part of the task gathered during Trial B (Figure 1, bottom). Such inappropriate alignment would lead to inappropriate estimation of inter-trial variation in signal magnitude. Our new assessment method uses software algorithms that address these limitations and allows for separate consideration of timing and signal magnitude variability, both of which may contain useful information with which to characterise variability in task performance.

This paper is the first to demonstrate the use of our methodology in characterising impaired upper limb motion during functional tasks. More specifically, we chose to investigate upper limb movement variability in chronic (stable) stroke patients and matched controls for a unilateral ('drinking') and bilateral ('moving a plate') functional task. Previous analysis of kinematic data collected with a 3D motion capturing system [5] as well as recent computer modelling [19] suggest that upper limb movement variability increases following stroke. We therefore hypothesize that stroke patients will exhibit increased movement variability as compared to healthy control subjects in constrained functional tasks, i.e. when the start/end point of the hand, and the sequence of events within the task, are both fixed and the object is picked up from and returned to a marked target position in each trial. Furthermore, we hypothesise that group differences in movement variability would persevere in a retest session 1 month after the initial test.

## Methods

### Subjects

Six stroke patients (Table 1) and six healthy control subjects were recruited from within Greater Manchester, UK, and gave written informed consent to participate in the study. Each control subject was matched in age, gender, and right/left hand dominance to his/her respective stroke patient. All subjects underwent a medical screening and corresponding descriptive data (Ashworth scale, Motricity Index, Light Touch Discrimination, Detection of Movement, Star Cancellation Test, Line Bisection Test) were collected. Control subjects showed no signs of central or peripheral nervous dysfunction. Stroke patients had to pass the star cancellation test and line bisection test to screen for visual neglect and visuospatial problems. All patients had to have sufficient residual hand opening and grasping ability on the affected side to be able to complete both functional tasks without assistance. Patients' scores with regard to tests of motor impairment, sensation, and spasticity are shown in Table 1.

**Table 1:** Descriptive parameters of stroke patients.

	Patient 1	Patient 2	Patient 3	Patient 4	Patient 5	Patient 6
Gender	Female	Male	Male	Male	Female	Male

Age	33	83	48	60	72	59

Dominant side	Right	Right	Right	Right	Right	Right

Affected Side	Right	Right	Left	Left	Right	Left

Time since stroke	3 years	4 years	3 years	6 months	3 years	2 years

Motricity Index*	66/100	76/100	76/100	63/100	76/100	76/100

Light Touch Discrimination*: Wrist, Hand	6/6, 6/6	6/6, 6/6	0/6, 4/6	3/6, 5/6	6/6, 6/6	5/6, 6/6

Movement Detection*: Shoulder, Elbow, Wrist, Thumb	6/6, 6/6, 6/6, 6/6	6/6, 6/6, 6/6, 6/6	6/6, 6/6, 3/6, 6/6	6/6, 6/6, 4/6, 4/6	6/6, 6/6, 6/6, 6/6	5/6, 6/6, 6/6, 6/6

Ashworth Scale*	1–2	1	1	3	1	0

Each control was matched in age, gender, and limb dominance to one patient.

*Hemiplegic Arm

### Experiment

The experimental protocol was approved by the UK Central Office of Research Ethics Committee (Ref. # 06/Q1405/7) and the University of Salford Research Governance and Ethics Committee (Ref. # RGEC05/28 and RGEC06/92). Subjects were asked to sit close up against a table and the position of the torso and the start/end point of the hands were marked on the cover of the table to allow for reproduction of a similar posture on the second test day. The location of each object, at a self-reported comfortable distance to the individual, was likewise marked on the table's cover. Care was taken that the object was placed within a distance that did not require engagement of the torso during task performance. Both tasks ('drinking from a glass' and 'moving a plate') were performed at a self-selected comfortable speed and involved a forward reach followed by hand opening and object grasp, object manipulation, and finally object release and arm retraction. Manipulation of the glass was composed of lifting it towards the mouth, holding it briefly, and then replacing the glass onto the table. Manipulation of the plate contained a small upwards lift of the plate in front of the torso, followed by a sideways translation of the plate towards the side where the plate was then lowered onto the table. Stroke patients performed the glass task with their affected arm, and controls had to use the same arm as their corresponding match. Furthermore, the plate was moved towards the affected side of the patient and this was copied by each corresponding control subject. Eight trials per task were recorded, and this was done on two different days, approximately 1 month apart.

### Instrumentation & data processing

**Figure 2:**
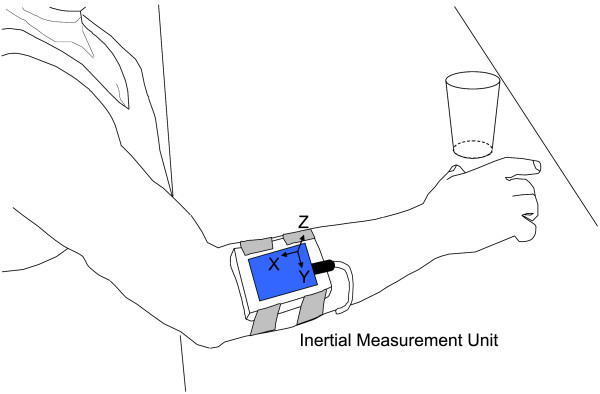
**Experimental set up**. The inertial sensor is shown on the proximal forearm as the subject reaches forward to grasp the glass.

**Figure 3:**
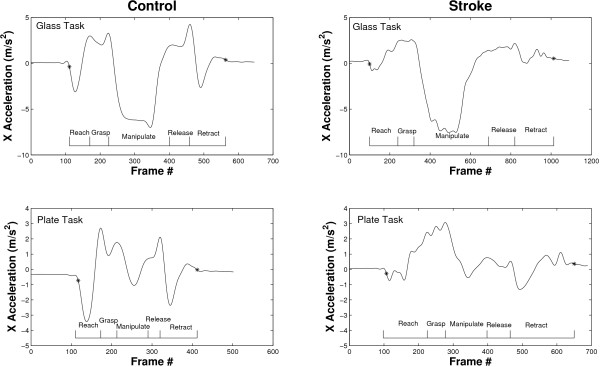
**Application of acceleration threshold algorithm**. Movement onset and termination indices are denoted by '*' and are superimposed onto the corresponding x acceleration trajectory. Sample plots are shown for a control subject (left) and stroke patient (right) for the glass task (top) and plate task (bottom).

An inertial sensor (Xsens Technologies B.V., Enschede, Netherlands) was placed on the forearm such that its x axis was roughly aligned with the forearm's longitudinal axis, pointing proximally, while the z axis was perpendicular to the forearm's surface, pointing upwards (Figure 2). Movement onset and termination of each trial were defined by an acceleration threshold algorithm (Matlab^®^) as the first and last frame where the x acceleration, roughly aligned with the longitudinal axis of the forearm, exceeded the mean resting value by ± 0.3 m/s^2^. For the definition of movement onset and end the acceleration signals were lowpass filtered with a 4^th ^order Butterworth filter and a cut-off frequency of 4 Hz. Figure 3 shows examples of acceleration trajectories and corresponding movement onset and termination indices for both tasks performed by a control subject and stroke patient. The derived indices were then used to truncate the original, unfiltered acceleration signals prior to their further processing with the variability software. Moreover, the movement time of each trial was defined as the time elapsed between these two frames and is reported as a secondary outcome measure.

### Definition of variability metrics

Inspired by recent work that addresses limitations with traditional approaches [17,20], we similarly employed dynamic programming [21] for curve-registration. The new approach presented here separately considers variability of any given signal in two parts, 1) variability in the timing of the signal, e.g. reoccurrence of a characteristic spike at a specific time instant in each trial, and 2) variability in the motion signal's magnitude, e.g. the maximum value of a characteristic spike reproduced from trial-to-trial. Our software algorithms, programmed in Matlab^®^, therefore uses a two stage process to quantify both aspects of movement variability separately.

**Figure 4:**
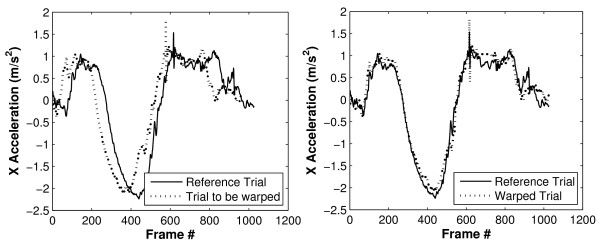
**Time warping of acceleration signals**. Linear acceleration signals of two trials that are to be investigated for trial-to-trial variability (left) and signals after having time-warped one signal to the declared reference (right).

The software first addresses the timing errors between trials before calculating differences in signal magnitude. Therefore, for each trial-to-trial comparison a reference trial (trial 1) is defined to which the other trial (trial 2) is "time-warped" (Figure 4). The variability in timing is then quantified by the amount of warping that was necessary to align the two trials. For each data point, p(t) = [x(t), y(t), z(t)] (a vector acceleration in 3 dimensional space), in trial 1, the software defines the 'error' between it and a given data point, p'(t') = [x'(t'), y'(t'), z'(t')], in trial 2 as the Euclidean distance between the two points:

$$d(p(t),p'(t'))=\sqrt{{\left(x(t)-x'(t')\right)}^{2}+{\left(y(t)-y'(t')\right)}^{2}+{\left(z(t)-z'(t')\right)}^{2}}$$

**Figure 5:**
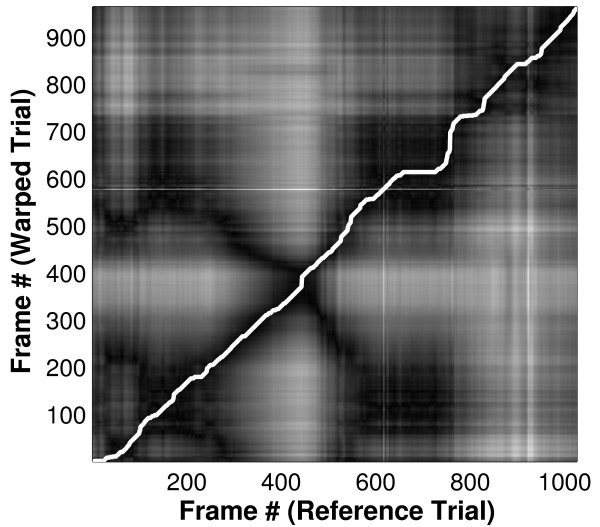
**Error surface and path of least error**. Error surface and path of least error produced when warping each frame of one trial to each frame of the reference trial. The axes represent time in trials 1 and 2 (i.e. t and t') respectively; light areas indicate a high error between points while dark areas indicate low error between points. Each frame represents 0.01 seconds.

Computing this error for every possible pairing of data points gives an error surface (Figure 5) in which the axes represent time in trials 1 and 2 (i.e. t and t') respectively; light areas indicate a high error between points (i.e. widely separated points) while dark areas indicate low error between points (i.e. points which are similar). Dynamic programming [21] is then used to calculate the path of minimum error (shown in white in Figure 5) across the diagonal of the error surface. This path defines the optimal time warping, f(t'), of trial 2 onto trial 1 and the RMS error between this path of least error and an ideal 45° line (f(t') = t+Δ, corresponding to a simple offset with no warping) represents the amount of time-warping done and is hereafter referred to as warping cost. The dynamic programming approach enforces the constraint that the warping does not change the temporal order of the data points in trial 2.

The variability in signal magnitude is then reflected by the RMS error between the reference trial and the warped trial. For each trial-to-trial comparison, RMS errors are obtained for time-warped x, y, and z accelerations and the average across all three axes is calculated.

Finally, the mean value across all trial-to-trial comparisons for a particular task on a particular day is calculated for each of the two variability metrics. The mean value of each metric (warping cost, RMS error) corresponding to each task (glass & plate task) is thereby determined for each subject on each day.

### Statistical Analysis

Paired t-tests [22] were used to compare stroke patients to matched controls for each task with regard to 1) mean warping cost (day 1 and 2, separately), 2) mean RMS error (day 1 & 2, separately), and 3) mean time to complete task (day 1 & 2, separately) and corresponding confidence intervals were determined. Furthermore, differences between stroke patients and corresponding matched controls were graphically visualized.

## Results

### Application of dynamic time warping

Only four trials per task per day were analyzed for the comparison of stroke patients to healthy controls. This was due to the stroke patients' insecure grasp of the object and onset of fatigue: trials during which the object was dropped were excluded and some patients fatigued so that no more than 4 good trials could be collected.

**Figure 6:**
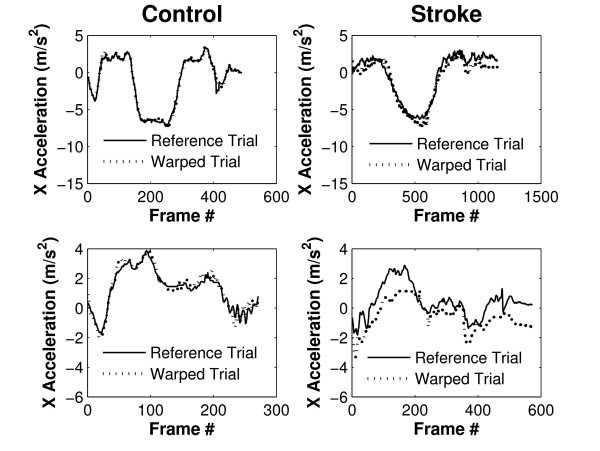
**X accelerations of two trials after time warping**. X acceleration signals of the glass task (top) and plate task (bottom) are shown for a control subject (left) and for a stroke patient (right).

Dynamic time warping successfully registered upper limb acceleration signals for both, stroke patients and controls. Figure 6 shows two acceleration signals per graph; one reference trial and one other comparison trial that has been time warped to align it with the reference trial. Graphs on the left show reference and time-warped acceleration signals of a healthy control subject for the glass task (top) and plate task (bottom). Graphs on the right are reference and time-warped trials of a stroke patient, again, for the glass task and plate task (top and bottom, respectively). Acceleration signals obtained from this stroke patient appeared less smooth as compared to the control subject, and this was to be expected given previous work that investigated movement smoothness in stroke patients [6]. Moreover, a larger RMS error between trials after time-warping can be observed for the stroke patient: compared to the control subject the stroke patient had an RMS error 2.9 times larger for the glass task and 3.5 times larger for the plate task.

### Warping cost and RMS error, glass task

**Figure 7:**
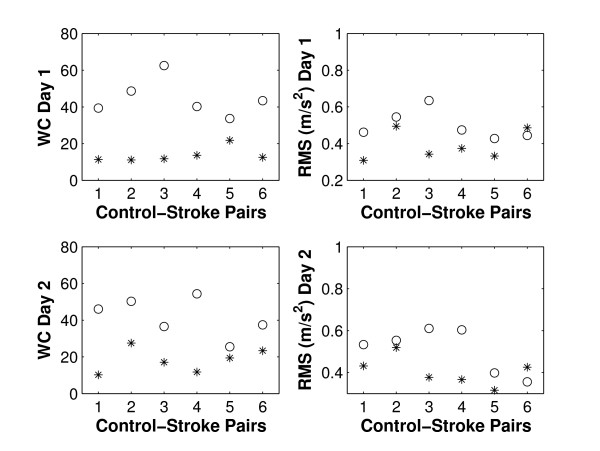
**Control-stroke-pairs, glass task**. Warping cost ('WC', left) and RMS error ('RMS', right) for day 1 and day 2 (top & bottom, respectively). Controls are denoted by '*' and stroke patients by 'o'.

Cost of warping was significantly different between stroke patients and controls for the glass task on both days (p = 0.002 and p = 0.008 for day 1 & 2, Table 2). A larger cost of warping was required to align trials of stroke patients, indicating that patients exhibited higher variability in timing of their motion than controls. Figure 7 illustrates that for both days all stroke patients exhibited higher variability in timing of the motion (as reflected in higher warping cost) than their corresponding control subject.

**Table 2:** Glass task variability metrics.

		Controls	Stroke	Significance	95% CI
Day 1	Warping Cost	13.71(4.06)	44.63(10.06)	p = 0.002	(-44.44, -17.42)
	
	RMS (m/s^2^)	0.39(0.08)	0.50(0.08)	p = 0.063	(-0.23, 0.009)

Day 2	Warping Cost	18.22(6.66)	41.71(10.58)	p = 0.008	(-37.76, -9.22)
	
	RMS (m/s^2^)	0.41(0.07)	0.51(0.11)	p = 0.086	(-0.23, 0.021)

Controls vs. stroke patients: group mean(group std). CI denotes 'confidence interval'.

RMS Error for the glass task had a p-value of less than 0.1 for both days but did not reach significance for the six stroke-control pairs (p = 0.063 and p = 0.086 for day 1 and day 2, respectively, see Table 2). Figure 7 shows the individual pairs (stroke and control): stroke patients showed higher variability in the accelerometer's signal magnitude (as reflected in a larger RMS error) in 5/6 cases on day 1 and day 2. The p-values for RMS error between groups became significant (p = 0.029 and p = 0.003) when the last pair was excluded from statistical analysis.

### Warping cost and RMS error, plate task

**Figure 8:**
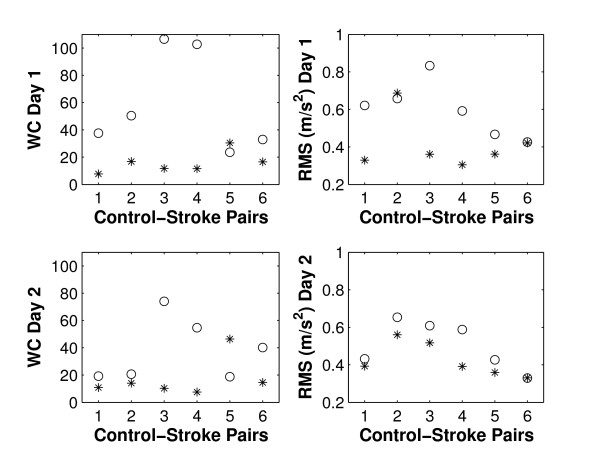
**Control-stroke-pairs, plate task**. Warping cost ('WC', left) and RMS error ('RMS', right) for day 1 and day 2 (top & bottom, respectively). Controls are denoted by '*' and stroke patients by 'o'.

For the plate task, warping cost did not reach significance when comparing stroke patients to controls (p = 0.050 and p = 0.180 for day 1 and 2, respectively, Table 3). Figure 8 illustrates that on both days 5/6 patients had larger cost of warping than their corresponding control subject.

**Table 3:** Plate task variability metrics.

		Controls	Stroke	Significance	95% CI
Day 1	Warping Cost	15.81(7.96)	58.96(36.45)	p = 0.050	(-86.35, 0.04)
	
	RMS (m/s^2^)	0.41(0.14)	0.60(0.15)	p = 0.064	(-0.39, 0.02)

Day 2	Warping Cost	17.29(14.48)	37.99(22.83)	p = 0.180	(-54.64, 13.43)
	
	RMS (m/s^2^)	0.43(0.09)	0.51(0.13)	p = 0.031	(-0.15, -0.01)

Controls vs. stroke patients: group mean(group std). CI denotes 'confidence interval'.

The RMS error was significant on day 2 (p = 0.031) and had a p-value of less than 0.1 on day 1 (Table 3). Figure 8 shows that 4/6 patients had a larger RMS error on day 1 than their corresponding control subject, and 5/6 did so on day 2.

### Time to complete functional tasks

Stroke patients took significantly more time when completing either of the functional tasks, and this was observed on both days (Table 4).

**Table 4:** Time (in seconds) to complete task.

		Controls	Stroke Patients	Significance	95% CI
Glass	Day 1	5.24(0.75)	12.30(2.45)	p = 0.001	(-9.85, -4.27)
	
	Day2	5.47(1.25)	11.47(2.16)	p = 0.006	(-9.30, -2.69)

Plate	Day1	3.86(1.11)	7.81(3.43)	p = 0.049	(-7.86, -0.04)
	
	Day2	3.59(0.73)	7.80(3.20)	p = 0.040	(-8.13, -0.29)

Controls vs. stroke patients: group mean(group std). CI denotes 'confidence interval'.

## Discussion

To our knowledge this is the first study that has applied dynamic time warping for curve registration to forearm acceleration signals from stroke patients and matched controls performing a unilateral and a bilateral functional task. It is noteworthy that two objective metrics of movement variability are obtained: 1) warping cost, representative of the variability in the timing of the acceleration signal, and 2) RMS error, representative of the variability in the signal's magnitude.

The warping cost for the glass task was significantly larger in stroke patients than controls on both days; since group differences persisted over the course of a month for this variability metric (as indicated by p-values < 0.05 on both days) it appears to be a promising clinical outcome measure if applied to unilateral functional tasks. It is noteworthy that we employed root mean square error calculation, a measure insensitive to trial length, to quantify warping cost. Moving generally at a slower speed therefore does not increase this metric, instead trial-to-trial variability of the timing of the acceleration signals' characteristics is captured by it. The RMS error for the glass task had a p-value < 0.1 when comparing stroke patients to controls and this became significant when the last stroke-control pair was removed from the analysis (p = 0.029 and p = 0.003 for day 1 and day 2, respectively). This parameter may therefore be useful in larger studies. The plate task provided less significant group differences when comparing variability measures for stroke patients to those of controls. This may be explained by the use of the healthy arm when moving the plate to the side: the affected arm may be guided and assisted by the healthy arm.

Curve registration was first applied to gait data by Sadeghi and colleagues [20]. They recognised that characteristic features, such as peak values, vary between individuals in their precise location within the gait cycle. Averaging time-normalised curves across individuals therefore results in loss of information. Sadeghi and colleagues used the technique of curve registration to more appropriately align subjects' gait data prior to further analysis. Because upper limb motions during functional tasks are not cyclic yet have repetitive characteristics if constrained, we decided to apply such an approach to upper limb acceleration signals and report the warping cost as a valuable outcome measure. Our results support this approach in that significant group differences with regard to time-warping were obtained. The next step is to apply this new methodology to a large number of stroke patients with various degrees of upper limb impairment and at different stages of rehabilitation to evaluate the merit of these metrics in routine clinical evaluations.

Stroke patients were more variable in their movement and needed more time to complete each task. Recent research investigated gait variability in conjunction with walking speed in young and older adults [23] and the authors concluded that increased gait variability in older adults is better explained by loss of strength and flexibility rather than slower walking speed. Similarly, future research needs to address the driving factors for upper limb movement variability in stroke and controls. Moreover, as with present research investigating gait variability [24,25], studies are needed to investigate the detailed interpretation of such data.

It is important to note that this work investigated group differences within a given day and showed if those differences persist when a retest is performed 1 month later. No direct trial-to-trial comparison between days was done, and it was therefore not necessary to exactly reproduce postural initial conditions and the sensor's orientation with respect to the forearm on the second test day.

In this initial study we acknowledge our small sample size and hence the wide confidence intervals. Nevertheless, graphical representation of stroke-control pairs for the glass task (Figure 7) supports application of variability metrics to unilateral functional tasks and encourages larger studies. In the long term, we envisage the design of a graphical user interface for the variability software that, together with an inexpensive and portable accelerometer, will allow researchers and clinicians to apply this software in routine clinical care.

In the future tasks could be subdivided into component features (e.g. reach forward, object grasp etc.) to provide further insight into particular aspects of upper limb function, for example hand opening, and their contribution to the variability scores. Moreover, given that task time is a crucial outcome measure in many clinical tests (e.g. ARAT), integration of variability measures with such tests merits further study. Furthermore, for evaluation of patients with no or poor hand opening, variability of other functional tasks could also be investigated. For example 'opening a door' and 'moving a box with both hands' are less challenging tasks and yet of real-life relevance and could accommodate patients with more severe impairments of the hand. Finally, there is recent evidence to show that, focusing on the characteristics of the movement itself rather than the outcome of the movement (e.g. end point accuracy in a pointing task), is of most benefit in promoting the recovery of normal motor patterns following stroke [26]. Currently, there is a limited range of tools for quantifying upper limb motion in the clinical environment [12], particularly with respect to performance of functional tasks, practice of which are viewed as key to the rehabilitation process. In our study we have introduced a new tool that allows for a detailed analysis of upper limb motion variability, measured with low cost sensors during performance of functional tasks. Further, the results presented here suggest that higher variability is associated with stroke. It is possible to speculate that such metrics could be used as part of a biofeedback tool to encourage a return to more normal levels of variability in performance of functional tasks. Further clinical trial work is required to explore whether a move towards more normal levels of variability is associated with a reduction in disability measures.

## Conclusion

The results of this study suggest that accelerometry, in conjunction with suitable variability metrics, has the potential to support clinicians and therapists in their assessment of upper limb impairment during functional task performance. Accelerometers are user-friendly and inexpensive and therefore of advantage in routine clinical evaluations.

## Competing interests

The authors declare that they have no competing interests.

## Authors' contributions

SBT designed the experiment, collected & analyzed data and drafted the manuscript. PAT, LPK and JS wrote/modified the variability software. DH and JYG made substantial contributions to conception of software design. CS and JR recruited stroke patients and collected descriptive data of patients and controls. All authors read and approved the final manuscript.
